# General-Purpose B-Mode Intraoperative Ultrasonography in Perirolandic Brain Metastasis Surgery: Practical Value and Limitations

**DOI:** 10.3390/brainsci16090920

**Published:** 2026-08-29

**Authors:** Yücel Doğruel, Hakkı Oğulcan Babacan, Doğan Gündoğan, Alper Tabanlı, Berk Burak Berker, Abuzer Güngör

**Affiliations:** 1 Department of Neurosurgery, Tepecik Training and Research Hospital, 35180 İzmir, Türkiye; babacanogulcan@gmail.com (H.O.B.); dogan.gundogan@yahoo.com (D.G.); alper_tabanli@hotmail.com (A.T.); 2 Department of Neurosurgery, Hatay Training and Research Hospital, 31030 Hatay, Türkiye; bburakberker@gmail.com; 3 Department of Neurosurgery, Faculty of Medicine, İstinye University, 34475 İstanbul, Türkiye; abuzergungor@gmail.com

**Keywords:** brain metastasis, perirolandic region, intraoperative ultrasonography, B-mode ultrasound, neuronavigation, extent of resection, eloquent cortex, image-guided neurosurgery

## Abstract

Background/Objectives: Perirolandic brain metastases are challenging to resect because small deviations from the surgical corridor may cause sensorimotor deficits. This study evaluated the stage-specific value and limitations of general-purpose, non-navigated B-mode intraoperative ultrasonography (iUS) with MRI-based frameless neuronavigation in a consecutive surgical cohort of perirolandic metastases. Methods: In this single-center, single-surgeon retrospective case series, 15 consecutive adults underwent microsurgical resection of perirolandic brain metastases using MRI-based frameless neuronavigation and a general-purpose B-mode ultrasound system, without functional mapping or neurophysiological monitoring. Extent of resection was assessed on contrast-enhanced MRI obtained 24 to 36 h after surgery, and post-resection cavity iUS findings were compared descriptively with early postoperative MRI. Results: Gross-total resection was achieved in 13 patients (86.7%). iUS modified intraoperative targeting in 6 of 15 patients: the entry point was repositioned after findings consistent with intraoperative brain shift in 4, and the lesion was localized in interhemispheric corridors in 2. Post-resection iUS suspected residual tissue in 7 of 14 patients, but only 1 corresponded to residual tumor on MRI; a thin remnant on the motor cortex was not identified as suspicious for residual tumor on iUS. A new or worsened motor deficit occurred in 2 patients (13.3%); at follow-up, this was persistent at 20 months in 1 and partially improved in the other. No patient required reoperation, and there was no 30-day mortality. Conclusions: General-purpose B-mode iUS aided exposure confirmation and trajectory refinement but had limited value for excluding thin residual tumor. When functional mapping or neurophysiological monitoring is unavailable, it may provide accessible real-time anatomical guidance; however, because it lacks functional information, it should not be considered an alternative to these functional adjuncts.

## 1. Introduction

Brain metastases (BM) occur in approximately 10–20% of adults with malignancy, and synchronous BM have an age-adjusted incidence of around 7.1 per 100,000 persons per year [1]. Surgical resection remains an important component of multidisciplinary management in selected patients, particularly for symptomatic, large, cystic, necrotic, hemorrhagic, or diagnostically uncertain lesions [2,3]. In the perirolandic region, however, surgery is especially demanding because the precentral and postcentral gyri and their adjacent sensorimotor networks support motor and somatosensory function of the trunk and extremities; even small deviations from the intended surgical corridor may result in clinically meaningful neurological deficits [3]. Resection in this region must therefore balance cytoreduction, edema relief, tissue diagnosis, and local disease control against the risk of sensorimotor morbidity [3,4]. The extent of resection remains clinically relevant, as gross-total resection has been associated with improved survival after surgery for BM [5].

Functional and image-guided adjuncts including intraoperative neurophysiological monitoring (IONM), cortical and subcortical stimulation mapping, awake craniotomy, navigated transcranial magnetic stimulation, diffusion tensor imaging, intraoperative MRI (iMRI), and fluorescence guidance have improved the safety of surgery in eloquent regions [4,6,7,8]. In specialized centers, these techniques have been associated with high resection rates and acceptable morbidity in perirolandic and central-region metastases [3,4]. However, they require dedicated equipment, trained personnel, infrastructure, and institutional experience that are not uniformly available; similar limitations related to equipment, operating-room logistics, personnel, and reimbursement may occur in many neurosurgical environments [9,10,11].

Intraoperative ultrasonography provides a practical real-time adjunct to preoperative image guidance. Frameless neuronavigation remains valuable for preoperative planning and initial orientation, but it relies on static preoperative imaging and may lose accuracy because of brain shift after positioning, dural opening, cerebrospinal fluid drainage, edema-related deformation, retraction, and progressive tumor removal [10,12]. B-mode intraoperative ultrasonography can partially address this limitation by providing repeatable, radiation-free imaging under current operative conditions [10,12]. It can be integrated into the surgical workflow at relatively low cost and without the logistical demands of intraoperative MRI [10]. Although the evidence base for intraoperative ultrasonography in brain metastasis surgery is less mature than that for gliomas [13], recent metastasis-specific data suggest that 2D intraoperative ultrasonography may improve the extent of resection compared with neuronavigation alone, particularly in settings where advanced adjuncts are not readily accessible [11].

Although the general capabilities and limitations of B-mode intraoperative ultrasonography are well established, its stage-specific use in perirolandic metastasis surgery has received limited dedicated study, particularly with general-purpose, non-navigated systems. In this region, intraoperative imaging must serve more than lesion localization alone. The operative window must be confirmed within this functionally critical region; the surgical trajectory may need to be adjusted in real time through narrow, deep, or interhemispheric corridors; and suspected residual tumor must be interpreted cautiously at the interface between the tumor and adjacent sensorimotor parenchyma. Available surgical series in this subgroup have primarily addressed functional mapping and neurophysiological monitoring [3,4]. In the present single-center retrospective study, we examined a consecutive series of patients with perirolandic BM treated with preoperative MRI-based frameless neuronavigation and general-purpose, non-navigated B-mode intraoperative ultrasonography, without functional mapping or neurophysiological monitoring. The study was designed to describe the extent of resection and early neurological outcomes observed with this workflow and, more specifically, to characterize the stage-specific role and limitations of B-mode iUS—from exposure confirmation and trajectory refinement, including cottonoid-guided orientation in transsulcal and interhemispheric corridors, to post-resection cavity assessment. Accordingly, B-mode iUS was evaluated as an anatomical adjunct to neuronavigation, not as a replacement for functional mapping or neurophysiological monitoring when these resources are available.

## 2. Materials and Methods

### 2.1. Study Population

This single-center retrospective observational case series included adult patients who underwent microsurgical resection of perirolandic brain metastases by a single senior surgeon (Y.D.) at the Department of Neurosurgery, Tepecik Training and Research Hospital, İzmir, Türkiye, between 1 June 2024 and 1 June 2026. The study was conducted in accordance with the Declaration of Helsinki and was approved by the institutional Ethics Committee of Tepecik Training and Research Hospital.

During the study period, 93 patients underwent microsurgical resection of brain metastases by the senior surgeon. All 93 cases were reviewed for anatomical eligibility. Fifteen patients (16.1%) fulfilled the perirolandic definition specified below, and all 15 were included in the study cohort; no surgically treated case meeting this definition was excluded. Inclusion criteria were age 18 years or older, histopathologically confirmed metastatic disease, perirolandic lesion location, resection performed using the workflow described below, and availability of adequate preoperative and early postoperative contrast-enhanced MRI. Patients were excluded if they underwent biopsy alone, lacked adequate early postoperative imaging, or underwent reoperation for a previously resected perirolandic metastasis. Patients with multiple brain metastases were eligible if the surgically treated target lesion fulfilled the perirolandic location criteria. In these patients, lesion-specific variables and extent-of-resection assessment referred to the operated perirolandic target lesion, whereas neurological and clinical outcomes were analyzed at the patient level.

### 2.2. Anatomical Definition and Data Collection

The perirolandic region was defined, in accordance with the microsurgical anatomy of the central lobe [14] and prior surgical series of central-region metastases [3], as the precentral and postcentral gyri, the central sulcus, and the paracentral lobule. A lesion was considered perirolandic when it involved any of these structures or was located in the subcortical white matter directly beneath them on preoperative contrast-enhanced MRI. Eligibility was therefore based on a structure-based anatomical definition; no distance threshold from the central sulcus or corticospinal tract was applied, and diffusion tensor imaging and functional MRI were not used as inclusion criteria. Lesion location was assessed on the preoperative MRI and neuronavigation dataset by H.O.B. and independently confirmed by B.B.B.; disagreements were resolved by consensus with the operating surgeon (Y.D.). Recorded variables included lesion side, volume, cortical or subcortical location, depth from the cortical surface, cystic or solid morphology, peritumoral edema, intended surgical corridor, presenting symptoms, preoperative motor strength using the Medical Research Council (MRC) scale, and Karnofsky Performance Status (KPS).

### 2.3. Surgical and Ultrasonographic Workflow

All patients underwent preoperative contrast-enhanced MRI according to the standard institutional protocol. Preoperative MRI-based frameless neuronavigation (Navient Image Guided Navigation System, ClaroNav, Toronto, ON, Canada) was used for craniotomy planning and initial intraoperative orientation. No patient underwent awake craniotomy, cortical or subcortical stimulation mapping, intraoperative neurophysiological monitoring, preoperative navigated transcranial magnetic stimulation, intraoperative MRI, fluorescence guidance, contrast-enhanced ultrasound, or navigated 3D ultrasound. Functional mapping and neurophysiological monitoring were unavailable for these procedures during the study period.

**Figure 1:**
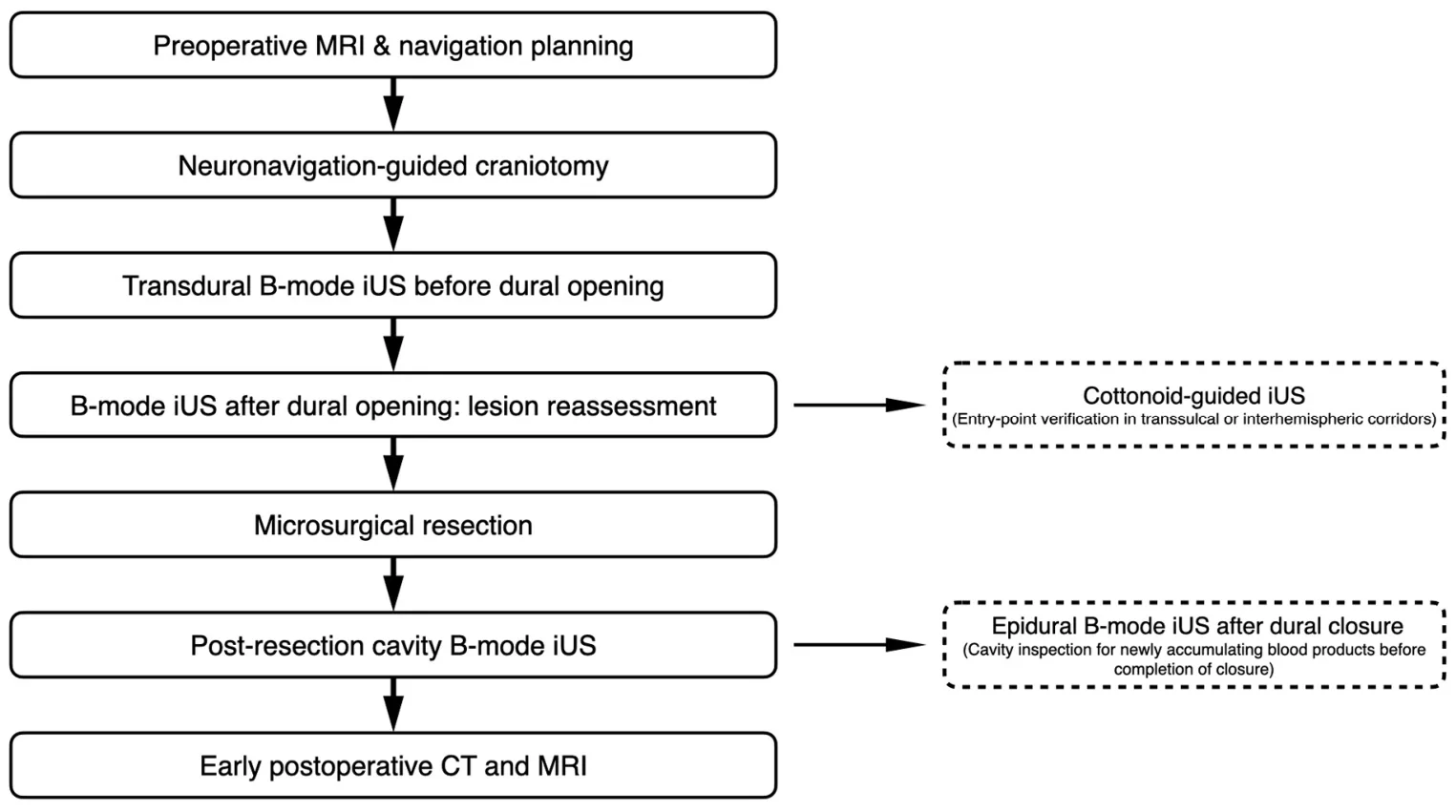
Surgical and ultrasonographic workflow for perirolandic brain metastasis resection. Additional ultrasonographic steps applied to defined subsets are shown separately (dashed boxes): cottonoid-guided iUS in all transsulcal and interhemispheric corridors, and epidural iUS after dural closure.

Intraoperative ultrasonography was performed using a general-purpose portable B-mode ultrasound system (SonoSite M-Turbo, FUJIFILM SonoSite Inc., Bothell, WA, USA) with a 13–6 MHz high-frequency linear transducer. The ultrasound system was a 2014-model unit operating with installed software version 51.80.110.011. Within the depth penetration range of the linear transducer, B-mode imaging was used to localize the lesion and adjacent anatomical landmarks. The ultrasound system was not integrated with the neuronavigation platform. Images were interpreted in real time by the operating surgeon without quantitative segmentation or volumetric analysis. The ultrasound workflow was categorized according to operative stage, as summarized in Figure 1.

**Figure 2:**
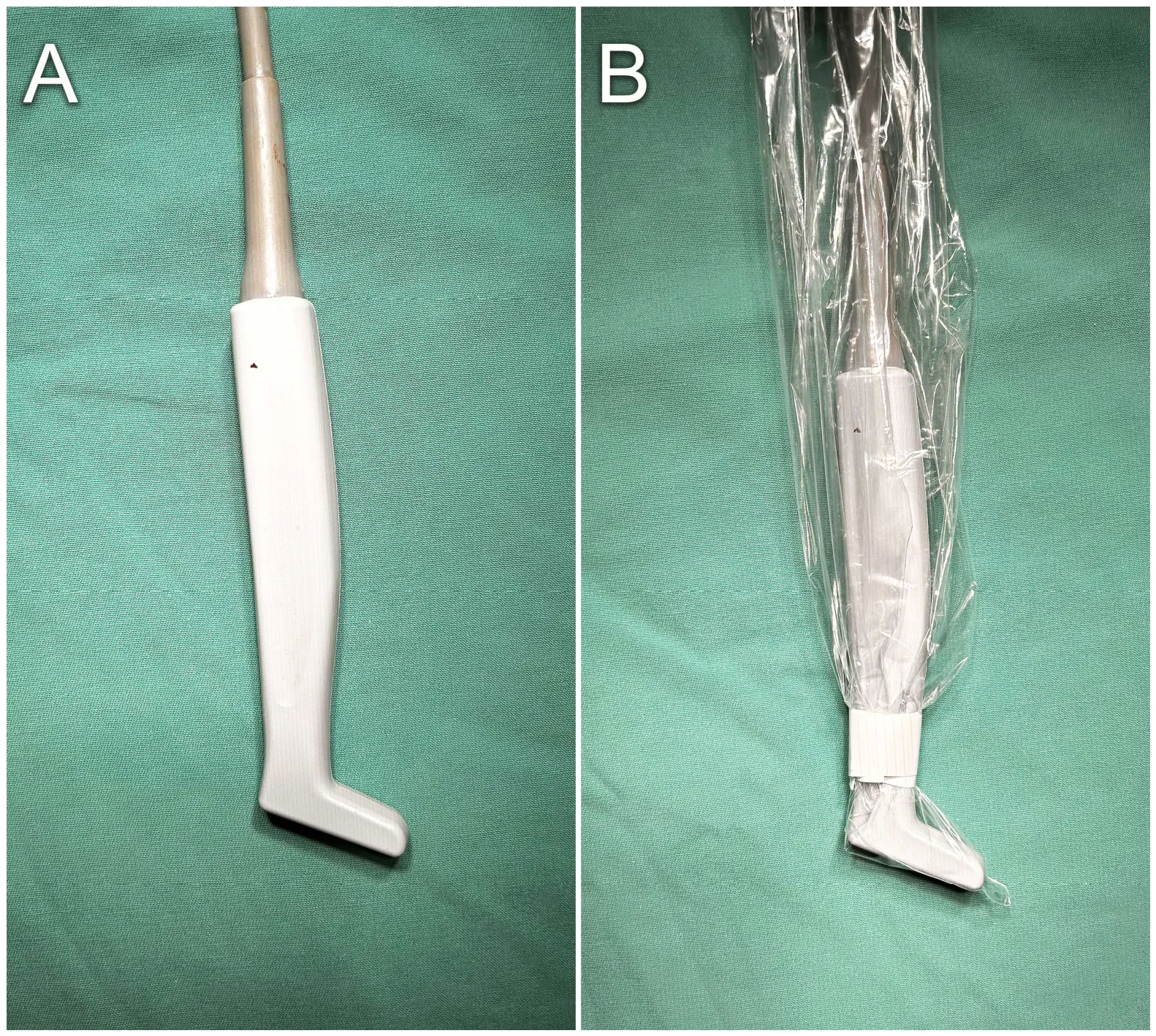
Sterile preparation of the general-purpose linear ultrasound transducer for intraoperative use. (**A**), High-frequency linear transducer before sterile covering. (**B**) The same transducer after placement of a sterile ultrasound probe sheath.

For intraoperative use, the linear transducer was covered with a sterile ultrasound probe sheath, and sterile coupling medium was used to maintain acoustic contact during transdural scanning, scanning after dural opening, post-resection cavity assessment, and epidural inspection after dural closure (Figure 2). Before placement within the sterile transparent sheath, ultrasound gel was applied directly to the transducer. During transdural scanning, the covered transducer was gently positioned directly on the dural surface; after dural opening, it was similarly positioned on the exposed brain surface without compression of the underlying tissue. For post-resection cavity assessment, the resection cavity was filled with sterile saline, and the covered transducer was positioned on the exposed brain surface adjacent to the cavity opening to visualize the cavity walls and surrounding tissue through the saline-filled cavity.

Before dural opening, a transdural scan was performed to confirm that the neuronavigation-guided craniotomy adequately covered the lesion. After dural opening, B-mode ultrasonography was repeated to reassess lesion position, depth, and orientation and to refine the intended cortical, sulcal, or interhemispheric trajectory. In all transsulcal or interhemispheric cases, cottonoid-guided ultrasonography was used as a real-time orientation adjunct for entry-point verification and trajectory definition: a saline-soaked cottonoid patty placed within the sulcus or along the interhemispheric corridor served as an echogenic marker on B-mode imaging, linking the visible operative field to the ultrasound view and confirming the orientation, depth, and progression of the corridor in relation to the lesion as dissection advanced [15,16]. The technique was not used for superficial lesions, in which the transducer could be positioned directly over the lesion and an intermediate echogenic marker was unnecessary; its use was therefore determined by corridor anatomy rather than case-by-case preference. Post-resection cavity ultrasonography was intended in all patients and was performed in all but one, in whom expedited closure was required because of intraoperative atrial fibrillation. Epidural ultrasound inspection after dural closure was performed as an exploratory adjunct to standard hemostasis. This step was introduced after mild resection-cavity blood products were observed on early postoperative CT for the third time during the study period. The rationale was that the interval required for watertight dural closure might allow newly accumulating cavity blood products to become detectable before completion of the operation; if identified, such a finding could prompt reopening of the dura and further hemostasis. Thereafter, post-closure epidural ultrasonography was performed in all subsequent patients except the patient requiring expedited closure. Post-resection and epidural ultrasonography were not part of a prospectively defined study protocol.

For retrospective analysis, an iUS-related change in intraoperative targeting was defined as either repositioning of the planned cortical or sulcal entry point on the basis of real-time ultrasonographic localization or reliance on iUS as the principal modality for lesion localization after intraoperative deformation. Navigation–anatomy discordance consistent with brain shift was recognized when a directly visible cortical landmark, such as a superficial vein or sulcus, no longer corresponded to its expected position on the neuronavigation dataset. Visible expansion of the cortical surface after dural opening was considered supportive of intraoperative deformation. Preoperative peritumoral edema, when present, was recorded as a potential contributing factor. In interhemispheric approaches, opening of the interhemispheric fissure was accompanied by visible lateral displacement of the retracted hemisphere. When this altered the relationship between the operative corridor and the preoperative navigation dataset, iUS was used to re-establish lesion localization and guide corridor progression. These assessments were qualitative, based on intraoperative observations documented contemporaneously in the operative notes, and extracted retrospectively; no numerical threshold for brain-shift distance or neuronavigation registration error was applied.

A craniotomy was tailored to the cortical projection of the lesion and intended surgical corridor. Cortical, sulcal, subpial, or interhemispheric corridors were selected according to lesion location and venous anatomy. Cortical, parasagittal, and bridging veins were systematically preserved. The decision to continue or terminate resection was based on visible residual tumor, surgical anatomy, hemostasis, and the presumed risk to adjacent sensorimotor tissue. B-mode ultrasonography was used as an adjunct for localization, exposure confirmation, and trajectory refinement; it was not used as a substitute for functional mapping or monitoring.

### 2.4. Outcome Assessment and Data Analysis

An early postoperative CT scan was obtained within the first postoperative hour to screen for acute hemorrhagic complications and to provide an initial assessment of the resection cavity. Postoperative blood products within the resection cavity were recorded when present. Early postoperative contrast-enhanced MRI, performed within 24 to 36 h after surgery, was used to assess the extent of resection. Early postoperative MRI was independently assessed by H.O.B. and D.G.; disagreements were resolved by consensus. Gross-total resection (GTR) was defined as the absence of visible residual enhancing tumor on early postoperative contrast-enhanced MRI. Non-gross-total resection (non-GTR) was defined as visible residual enhancing tumor.

Neurological status was assessed preoperatively, in the immediate postoperative period, and at discharge. Motor strength was documented using the MRC scale. A new or worsened postoperative motor deficit was defined as a new motor deficit or a decrease of at least one MRC grade compared with the preoperative examination. Postoperative seizures, reoperation, 30-day mortality, and length of hospital stay were recorded. Details of postoperative oncological treatment were not analyzed. Clinical follow-up duration was calculated from the date of surgery to the last documented outpatient or oncology visit. Available follow-up examinations were reviewed for motor status using the MRC scale, and KPS and vital status at the last clinical follow-up were recorded descriptively.

Clinical, radiological, operative, and follow-up data were extracted retrospectively from institutional records and anonymized before analysis. Given the descriptive nature of the study and the small sample size, no a priori sample size calculation was performed, and no formal hypothesis testing was undertaken. Post-resection cavity ultrasonography findings were compared with early postoperative MRI at the patient level in the 14 patients in whom it was performed. An iUS examination was classified as positive when residual tumor was suspected along the cavity wall and as negative when no residual tumor was suspected. Early postoperative contrast-enhanced MRI was used as the reference standard, with visible residual enhancing tumor classified as a positive MRI finding. A 2 × 2 contingency table was constructed. Because only 2 patients had residual enhancing tumor on early postoperative MRI, this comparison was treated as exploratory and was reported descriptively; derived diagnostic measures were provided with 95% confidence intervals (Wilson score method) for completeness only. Categorical variables were reported as counts and percentages, and continuous variables as medians with ranges. No comparative subgroup analysis was performed because ultrasound steps were not allocated prospectively and the study was not powered for such comparisons.

Because all patients undergoing resection of perirolandic metastases by the senior surgeon during the study period were managed using the described neuronavigation-plus-B-mode ultrasonography workflow, no contemporaneous perirolandic surgical cohort treated without intraoperative ultrasonography was available. The non-perirolandic metastasis cases operated on during the same period were not considered an appropriate comparator because of substantial anatomical and surgical heterogeneity.

## 3. Results

### 3.1. Patient and Lesion Characteristics

Fifteen patients met the inclusion criteria. Patient and lesion characteristics are summarized in Table 1. The median age was 62 years (range, 33–79 years), and the cohort included 10 men and 5 women. The primary tumor was lung cancer in 10 patients, breast carcinoma in 2, gastrointestinal or colorectal carcinoma in 2, and endometrial carcinoma in 1. Eight patients had a solitary brain metastasis, whereas 7 had multiple brain metastases. The operated target lesion was right-sided in 9 patients and left-sided in 6.

All target lesions involved the perirolandic region. The lesion was located in the precentral gyrus in 9 patients, the postcentral gyrus in 3, and the paracentral lobule in 3. The median target tumor volume was 8.13 cm^3^ (range, 2.23–55.94 cm^3^). The median preoperative Karnofsky Performance Status was 90 (range, 50–100). A preoperative motor deficit was present in 11 patients (73.3%), and 4 patients (26.7%) had preoperative seizures.

### 3.2. Intraoperative Ultrasonographic Workflow

Transdural B-mode ultrasonography before dural opening and repeat B-mode ultrasonography after dural opening were performed in all 15 patients (100.0%). The intraoperative ultrasonographic workflow and extent of resection data are summarized in Table 2. The transdural scan confirmed that the neuronavigation-guided craniotomy covered the target lesion in all cases, and no secondary enlargement of the bone opening was required. After dural opening, B-mode ultrasonography was used to reassess lesion position, depth, and orientation under current operative conditions before cortical, sulcal, or interhemispheric entry.

Cottonoid-guided ultrasonography was used in 5 patients (33.3%), comprising all transsulcal (*n* = 3) and interhemispheric (*n* = 2) approaches in the series. Post-resection cavity ultrasonography was performed in 14 patients (93.3%) to inspect the cavity for obvious residual solid tumor or gross cavity abnormality. In the remaining patient, atrial fibrillation developed early during surgery and persisted; after tumor removal, rapid closure was performed, and post-resection cavity ultrasonography was not performed. Epidural ultrasonographic inspection after dural closure was performed in 5 patients (33.3%) as an exploratory adjunct to standard hemostasis. No post-closure ultrasonographic finding led to reopening of the dura or additional hemostatic intervention.

Intraoperative B-mode findings modified the intraoperative targeting in 6 of 15 patients (40.0%). In 4 patients, real-time B-mode ultrasonography revealed a mismatch between the neuronavigation-defined entry point and the current ultrasonographic lesion location, consistent with intraoperative brain shift, and the entry point was repositioned accordingly before cortical or sulcal incision. In the 2 interhemispheric cases, opening of the interhemispheric fissure was associated with visible lateral displacement of the retracted hemisphere and loss of reliable correspondence with the preoperative navigation dataset, and the lesion was localized primarily with B-mode ultrasonography to guide resection. In addition, post-resection cavity B-mode imaging raised suspicion of thin capsular residual tissue along the cavity wall in 6 patients; in all 6, direct microsurgical inspection confirmed no resectable residual tumor, resection was terminated, and gross-total resection was subsequently confirmed on early postoperative MRI.

### 3.3. Extent of Resection

**Figure 3:**
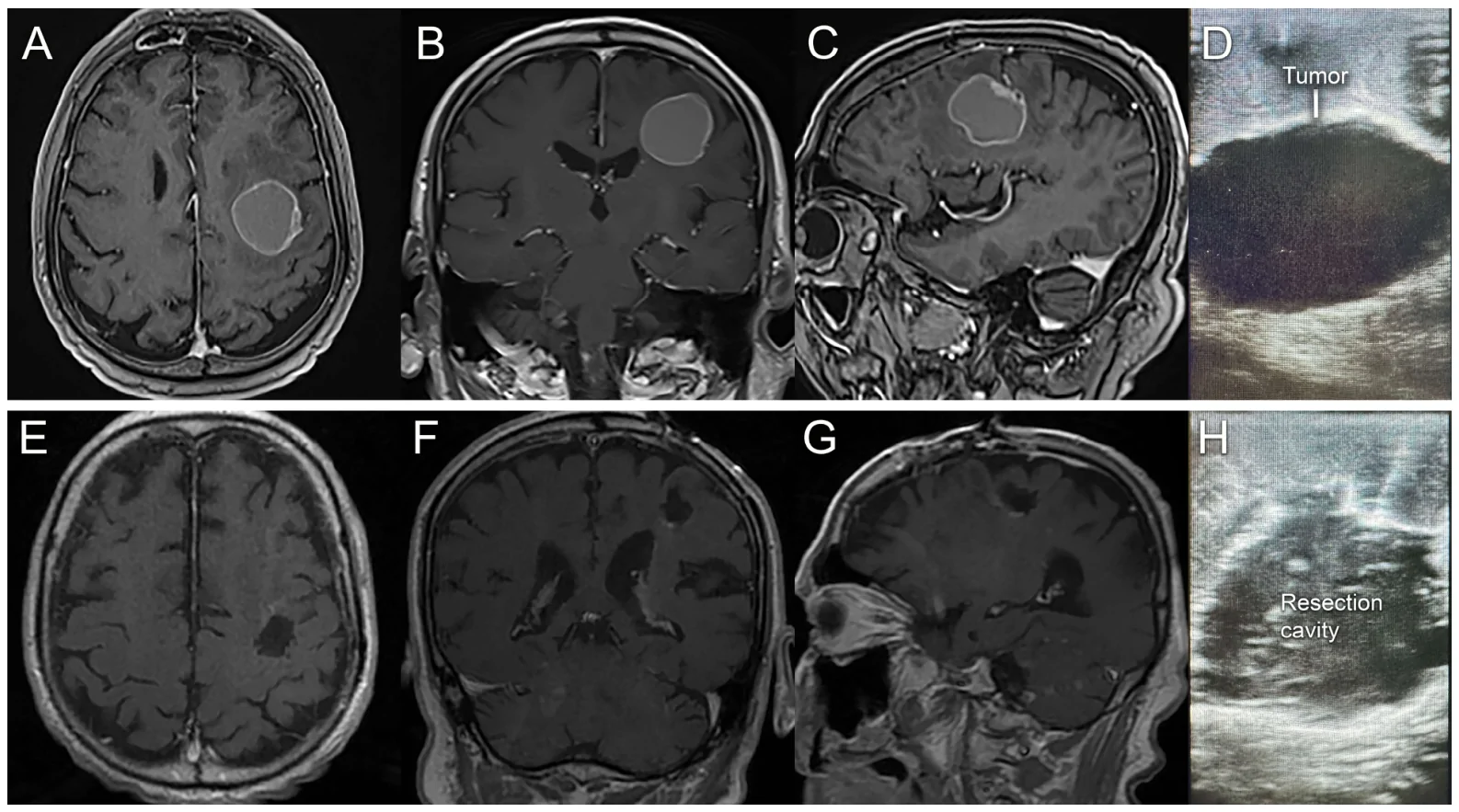
Illustrative case of a cystic perirolandic brain metastasis. (**A**–**C**) Preoperative contrast-enhanced MRI showing the cystic perirolandic lesion on axial, coronal, and sagittal images. (**D**) B-mode intraoperative ultrasonography after dural opening demonstrating the lesion before tumor removal. The tumor is labeled directly in the ultrasonographic image. (**E**–**G**) Early postoperative contrast-enhanced MRI on axial, coronal, and sagittal images confirming gross-total resection. (**H**) Post-resection cavity B-mode ultrasonography. The resection cavity is labeled directly in the ultrasonographic image. In (**D**,**H**), the ultrasound probe was positioned in an approximately sagittal plane.

**Figure 4:**
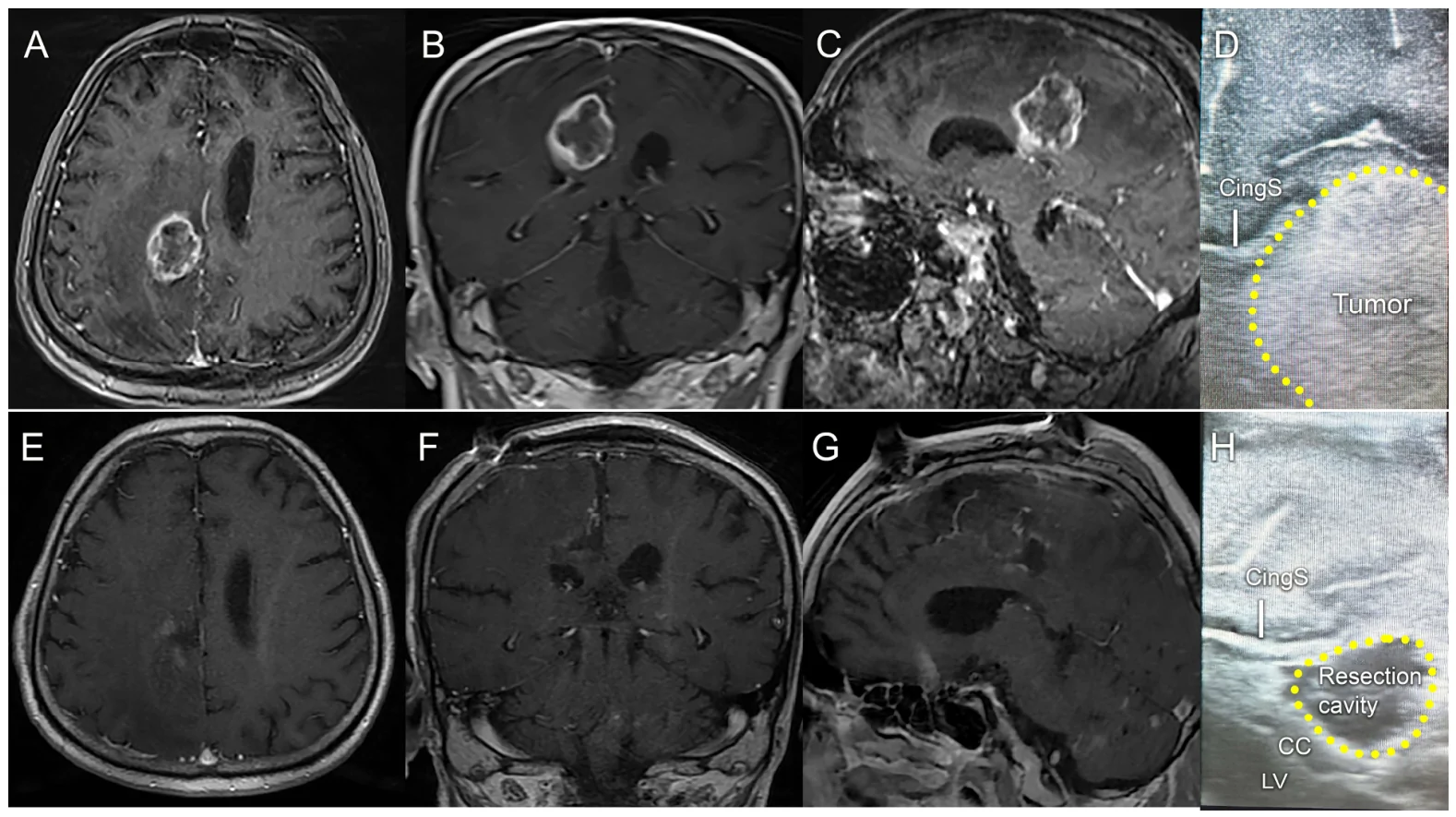
Illustrative case of a solid perirolandic brain metastasis. (**A**–**C**) Preoperative contrast-enhanced MRI showing the solid perirolandic lesion on axial, coronal, and sagittal images. (**D**) B-mode intraoperative ultrasonography after dural opening demonstrating the lesion before tumor removal. The tumor is delineated by the yellow dotted outline. (**E**–**G**) Early postoperative contrast-enhanced MRI on axial, coronal, and sagittal images confirming gross-total resection. (**H**) Post-resection cavity B-mode ultrasonography. The resection cavity is delineated by the yellow dotted outline. In (**D**,**H**) the ultrasound probe was positioned in an approximately sagittal plane. CingS, cingulate sulcus; CC, corpus callosum; LV, lateral ventricle.

Early postoperative contrast-enhanced MRI demonstrated gross-total resection in 13 patients and non-gross-total resection in 2. Therefore, the GTR rate was 86.7%, and the non-GTR rate was 13.3%. Representative cases achieving gross-total resection are illustrated in Figure 3 and Figure 4, including preoperative and early postoperative contrast-enhanced MRI and corresponding intraoperative B-mode ultrasonographic images. In both non-GTR cases, resection was intentionally limited to avoid unacceptable neurological or vascular morbidity. In one patient, a capsular remnant adherent to the primary motor cortex was left in situ to preserve motor function. In the other patient, a firm residual fragment adherent to the superior sagittal sinus wall could not be safely removed despite ultrasonic aspiration and was deliberately left in place to avoid venous injury.

### 3.4. Post-Resection Cavity Ultrasonography Compared with Early Postoperative MRI

Post-resection cavity B-mode ultrasonography was performed in 14 patients, and its findings were compared with early postoperative contrast-enhanced MRI as the reference standard (Table 3). B-mode imaging suggested residual tissue along the cavity wall in 7 patients, of whom only 1 had residual tumor confirmed on MRI; the remaining 6 were classified as gross-total resection, representing false-positive capsular signals. Among the 2 patients with residual tumor on MRI, B-mode ultrasonography identified the residual lesion in 1 (a firm fragment adherent to the superior sagittal sinus) but did not identify the other, a thin capsular remnant on the primary motor cortex, as suspicious for residual tumor. This remnant was recognized on direct microsurgical inspection and deliberately left in situ to preserve motor function, but it was not distinguishable on B-mode imaging.

Because only 2 patients had MRI-confirmed residual tumor, formal diagnostic-performance measures could not be estimated with meaningful precision. Accordingly, this comparison was interpreted descriptively and should be regarded as exploratory rather than as a definitive assessment of diagnostic accuracy; the derived measures with 95% confidence intervals are provided in the footnote to Table 3 for completeness only.

### 3.5. Early Postoperative Imaging and Clinical Outcomes

Early postoperative CT demonstrated mild blood products within the resection cavity in 3 patients (20.0%). These findings were not associated with neurological deterioration or reoperation. Early postoperative imaging and clinical outcomes are summarized in Table 4.

A new or worsened postoperative motor deficit occurred in 2 patients (13.3%). Motor status was improved or partially improved compared with the preoperative examination in 5 patients (33.3%) and remained stable without new worsening in 8 patients (53.3%). Overall, 13 of 15 patients (86.7%) had no postoperative motor worsening. The median clinical follow-up was 6.1 months (range, 1.1–20.2 months), and all patients had at least 1 month of clinical follow-up. Ten of 15 patients were alive at last follow-up. The median KPS at last clinical follow-up was 80 (range, 50–100). Of the 2 patients who developed a new or worsened postoperative motor deficit, one showed partial improvement within the first postoperative month but died on postoperative day 107 before further neurological assessment; in the other, the deficit persisted at the most recent documented motor assessment, at 20 months.

Early postoperative seizures occurred in 2 patients (13.3%), including one generalized tonic-clonic seizure. Both patients with early postoperative seizures were controlled with antiepileptic medication and were seizure-free at follow-up. No patient required reoperation. There was no 30-day mortality. The median length of hospital stay was 6 days (range, 5–14 days).

## 4. Discussion

In this single-center, single-surgeon series of perirolandic brain metastases, preoperative MRI-based frameless neuronavigation combined with general-purpose B-mode intraoperative ultrasonography was used as a practical image-guided workflow in a high-risk anatomical region. Gross-total resection was achieved in 13 of 15 patients, whereas non-gross-total resection was observed in 2. A new or worsened postoperative motor deficit occurred in 2 patients, while 13 had no postoperative motor worsening. No patient required reoperation, and no 30-day mortality was observed. These outcomes are descriptive; in the absence of a comparator group, they cannot be attributed to intraoperative ultrasonography. The main contribution of B-mode ultrasonography in this series was not the exclusion of small residual tumor, but real-time confirmation of the operative window and refinement of the surgical trajectory under current intraoperative conditions.

Previous studies have compared 2D intraoperative ultrasonography with neuronavigation alone in broader brain-metastasis cohorts [11], characterized navigated 3D ultrasound in metastasis surgery [17], and described cottonoid-guided ultrasonographic orientation across intracranial pathologies [15]. The present series provides a stage-specific characterization of how a general-purpose, non-navigated B-mode system performed in an anatomically defined, consecutive cohort of perirolandic metastases. Such characterization is particularly relevant here, where both small targeting errors and attempts to resolve equivocal cavity-wall findings adjacent to functional cortex may carry clinically meaningful functional consequences.

The clearest practical contributions of B-mode iUS occurred before and during lesion access. B-mode findings altered intraoperative targeting in 6 of 15 patients: in 4, they prompted repositioning of the entry point when real-time ultrasonographic lesion localization no longer corresponded with the preoperative navigation dataset; in the 2 interhemispheric cases, iUS became the principal modality for lesion localization after lateral displacement of the hemisphere during retraction. The cottonoid-guided iUS technique was used as an established adjunct [15] to maintain orientation in transsulcal and interhemispheric corridors.

By contrast, post-resection cavity assessment was less reliable: only 1 of 7 cavity-wall findings suspicious for residual tissue corresponded to residual tumor on early postoperative MRI, and a true thin remnant on the primary motor cortex was not identified as suspicious for residual tumor on iUS. These findings define a stage-specific utility profile: general-purpose B-mode ultrasonography appears most useful as a real-time anatomical adjunct for exposure and trajectory control, whereas cavity-wall findings require cautious interpretation and cannot substitute for early postoperative MRI.

Perirolandic metastases remain difficult lesions to operate on because the margin for error is small. This region supports motor and somatosensory function, and injury to cortical or subcortical sensorimotor structures may result in clinically meaningful deficits [3]. Dedicated surgical literature in this subgroup has largely emphasized function-guided approaches, including intraoperative brain mapping and neurophysiological monitoring [4]. Awake mapping and preoperative navigated transcranial magnetic stimulation have also been used to improve planning and functional safety in selected motor-eloquent lesions, including metastases [6,7,8]. Studies of tumors involving the primary motor cortex further illustrate that even with stimulation mapping, surgical decision-making remains constrained by the risk of motor morbidity and the functional relevance of the involved cortex [18,19]. The present series describes the early surgical and radiological outcomes observed with an image-guided workflow during a period in which functional mapping and neurophysiological monitoring were unavailable for these procedures. In such circumstances, including resource-limited settings, B-mode iUS may provide real-time anatomical guidance, but it does not provide functional information and should not be considered an alternative to functional mapping or neurophysiological monitoring when these adjuncts are available.

The first useful role of B-mode iUS was confirmation of the surgical exposure. A transdural scan was performed in all patients after the neuronavigation-guided craniotomy and before dural opening. In every case, the scan confirmed that the lesion was within the operative window, and no secondary enlargement of the bone opening was required. This step is simple, but it is valuable in perirolandic surgery. A small or eccentric craniotomy may force a less favorable approach toward eloquent cortex, whereas an unnecessarily wide exposure may increase manipulation of functional cortical territory. The transdural scan provided a quick intraoperative check that the planned exposure matched the actual target before the surgeon proceeded to dural opening and cortical or sulcal entry.

The second and probably more important role of B-mode iUS was trajectory refinement. Frameless neuronavigation is useful for planning and initial orientation, but it remains linked to preoperative imaging. Progressive intraoperative deformation—from dural opening and cerebrospinal fluid drainage through retraction and tumor removal—can reduce its accuracy, a recognized limitation of image-guided neurosurgery [10,17,20]. This limitation is relevant in perirolandic surgery, where a few millimeters of deviation may alter the risk profile of a cortical or sulcal entry point. Repeated B-mode scanning after dural opening allowed the surgeon to reassess lesion position, depth, and orientation before entering the cortex, sulcus, or interhemispheric corridor. In this role, iUS acted as a real-time anatomical update rather than as a replacement for functional information. The value of iUS as a real-time adjunct for lesion localization and resection control has also been reported in broader brain mass lesion series [12].

Cottonoid-guided ultrasonography was used in all 5 patients treated through transsulcal (*n* = 3) or interhemispheric (*n* = 2) corridors. In these cases, cottonoid patties served as echogenic markers linking the visible operative field to the ultrasound image. This allowed real-time delineation of the entry point and surgical trajectory. Its value in this series was practical: it made non-navigated 2D ultrasound easier to interpret during narrow or deep approaches. This use is consistent with the cottonoid-guided iUS concept described previously [15]. The present series documents its use as part of the broader stage-specific B-mode workflow. Because the technique was applied to a small, anatomically defined subgroup, no conclusion can be drawn regarding its independent effect on the extent of resection or neurological outcome.

Post-resection cavity ultrasonography was performed in 14 patients, but residual assessment was the least reliable part of the workflow. B-mode iUS could help identify obvious solid residual tissue or gross cavity abnormality. It was less reliable for thin capsular, rim-like, or sheet-like residual tissue along the cavity wall. In cystic metastases, after decompression of the cystic component, the collapsed wall could be difficult to distinguish from reactive tissue, blood products, or normal parenchyma. This limitation is important. In the present workflow, the decision to stop resection was based on direct microsurgical inspection, surgical anatomy, hemostasis, and the presumed risk to adjacent sensorimotor tissue; postoperative contrast-enhanced MRI remained the standard for final assessment of extent of resection. This interpretation is in line with the broader iUS literature, which emphasizes both the value of real-time imaging and the influence of image quality, artifacts, blood products, air, hemostatic material, and operator experience on interpretation [10,13]. This is also supported by diagnostic-property data showing good specificity but limited sensitivity of intraoperative ultrasound for small residual tumor, particularly the last milliliter of disease [21]. In the present series, this limitation was examined directly. Using early postoperative MRI as the reference standard, post-resection B-mode ultrasonography detected residual tumor in only 1 of 2 patients with confirmed residual disease. In 6 patients, B-mode imaging suggested capsular residual tissue that was confirmed neither on microsurgical inspection nor on postoperative MRI, whereas the single thin capsular remnant left on the primary motor cortex was not identified as suspicious for residual tumor on iUS. Given the small number of MRI-positive cases, these observations are exploratory and do not permit a definitive estimate of diagnostic accuracy; nevertheless, they indicate that general-purpose B-mode iUS is not a reliable means of excluding thin residual tumor along the cavity wall, and that early postoperative MRI remains necessary for definitive extent-of-resection assessment.

The present experience is also consistent with recent brain metastasis-specific data. Sirbu and colleagues reported a higher GTR rate with 2D iUS than with standard neuronavigation alone in a matched cohort of patients undergoing brain metastasis resection [11]. Their study supports the role of 2D iUS as an accessible real-time adjunct in metastasis surgery. The present series adds a perirolandic-specific perspective: in this region, the most useful contribution of general-purpose B-mode iUS may be trajectory control and exposure verification rather than fine residual detection. This distinction is central to the message of this study. Surgeons using a general-purpose, non-navigated B-mode system may find it useful for craniotomy confirmation, lesion localization, depth estimation, and corridor orientation. They should not rely on it to exclude thin residual tumor at the cavity wall.

The 2 non-GTR cases in this study illustrate the onco-functional balance required in this anatomical region. In one patient, a capsular remnant adherent to the primary motor cortex was left in place to preserve motor function. In the other patient, a firm residual fragment adherent to the superior sagittal sinus wall could not be safely removed despite dissection and ultrasonic aspiration and was deliberately left in place to avoid venous injury. These cases underscore that maximal safe resection in the perirolandic region is not defined by imaging ambition alone. It also depends on functional cortex, venous anatomy, tumor consistency, and the surgeon’s judgment regarding the risk of irreversible morbidity. The importance of the extent of resection in brain metastasis surgery is supported by clinical outcome data, but in eloquent regions, this objective must be balanced against functional preservation and surgical safety [3,5,18].

Post-closure epidural ultrasonography was introduced as an exploratory step after a third occurrence of mild resection-cavity blood products was identified on early postoperative CT during the study period. The underlying rationale was that the interval required for watertight dural closure might allow newly accumulating cavity blood products to become detectable before completion of the operation, potentially permitting dural reopening and additional hemostasis. It was subsequently performed in 5 patients, comprising all remaining cases except one, in whom expedited closure was required because of intraoperative atrial fibrillation. No examination identified a finding that prompted dural reopening or further hemostatic intervention. The closed dura did not produce an obvious loss of image interpretability in these cases, although image quality was not formally compared with open-dura imaging, and possible deep-field reverberation or attenuation was not systematically assessed. Because no subsequent clinically relevant cavity hemorrhage occurred, the ability of post-closure ultrasonography to detect such bleeding could not be evaluated. Its potential value should therefore be assessed prospectively using a standardized protocol.

The early neurological findings describe the postoperative functional profile of this consecutive surgical cohort. Eleven patients had a preoperative motor deficit. Postoperatively, motor function improved or partially improved in 5 patients and remained stable without new worsening in 8. A new or worsened motor deficit occurred in 2 patients. These findings should not be interpreted as evidence that iUS reduced neurological morbidity, nor as an indication that surgery in this region can be undertaken without functional monitoring where such monitoring is available. Functional preservation depends on patient selection, lesion anatomy, surgical corridor, venous anatomy, tumor consistency, and intraoperative judgment. Without mapping or IONM, B-mode iUS provides anatomical information only.

Postoperative oncological management remains essential after brain metastasis resection, although it was not the focus of the present study. Randomized data have established the role of postoperative radiation strategies for intracranial control, including whole-brain radiotherapy and cavity stereotactic radiosurgery [22,23,24]. In the present series, postoperative oncological treatment was determined outside the study protocol, and oncological outcomes were not analyzed. The study was designed to evaluate intraoperative workflow and early surgical outcomes rather than local control or survival.

The limitations of cavity-wall assessment observed in this series relate to the specific general-purpose B-mode system used and should not be generalized to intraoperative ultrasonography as a whole. The B-mode acquisition used here provided grayscale structural information without additional characterization of tissue stiffness or perfusion. Advanced ultrasonographic techniques may therefore perform differently. Shear wave elastography provides complementary information based on tissue stiffness and was associated with a higher observed sensitivity than conventional B-mode imaging for residual tumor detection in a heterogeneous brain-tumor cohort [25]. Contrast-enhanced ultrasonography can provide perfusion-based tissue contrast and has been used to improve characterization of suspected residual tumor [26]; preliminary metastasis-specific data also suggest a potential role in delineating lesions with poorly defined conventional ultrasonographic margins [27]. Dedicated small-footprint intraoperative probes, which can be introduced into the resection cavity to scan its walls directly rather than imaging through the adjacent cortical surface, have been used for remnant detection in high-grade intracerebral tumors including metastases [28], whereas Doppler techniques can provide complementary vascular information [10]. Whether any of these approaches can address both the false-positive cavity-wall signals and the failure to identify a thin capsular remnant remains to be determined prospectively in perirolandic metastases.

Several limitations should be acknowledged. This retrospective, single-center, single-surgeon series included only 15 patients and lacked a contemporaneous control group, since all perirolandic metastases resected during the study period were managed using the described workflow; causal inference regarding the effect of iUS on resection or neurological outcomes is therefore precluded. Functional mapping and neurophysiological monitoring were unavailable during the study period; the findings describe outcomes under this constraint and should not be interpreted as supporting B-mode iUS in place of functional adjuncts when available. Referral and treatment-selection bias cannot be excluded because the number of perirolandic metastases managed non-surgically was unavailable, limiting generalizability beyond this surgical cohort. The ultrasound workflow was not prospectively standardized; post-resection cavity iUS was completed in 14 of 15 patients, whereas epidural iUS was introduced during the study period and was not applied throughout the cohort. The general-purpose, non-navigated B-mode system reflects the accessibility-focused nature of the workflow but limits precision and reproducibility. Brain shift and navigation–anatomy discordance were therefore assessed qualitatively using operative landmarks and contemporaneous operative notes rather than quantitative measurements of displacement or registration error. Sensory outcomes were not assessed with a standardized scale, and clinical follow-up was heterogeneous, with neurological assessments not performed at uniform postoperative time points. Formal analyses of local control and survival were not performed because the study focused on intraoperative workflow and early surgical outcomes.

## 5. Conclusions

In this single-center, single-surgeon series, 15 patients with perirolandic brain metastases were treated using MRI-based frameless neuronavigation and general-purpose, non-navigated B-mode intraoperative ultrasonography. Gross-total resection was achieved in 86.7% of cases; there was no 30-day mortality, and 86.7% of patients had no new or worsened postoperative motor deficit. These outcomes are descriptive and cannot be attributed to intraoperative ultrasonography in the absence of a comparator group. The principal contributions of B-mode iUS were stage-specific: confirming adequate craniotomy coverage before dural opening, refining the surgical trajectory under current intraoperative conditions, and facilitating cottonoid-guided orientation in transsulcal or interhemispheric approaches. By contrast, post-resection cavity-wall assessment was less reliable for detecting thin residual tumor and was prone to false-positive capsular or artifactual signals, although these observations remain exploratory given the small number of MRI-confirmed residual tumors. General-purpose B-mode iUS should therefore be regarded as an accessible real-time anatomical adjunct to neuronavigation, but not as a substitute for functional mapping, neurophysiological monitoring, or early postoperative MRI. Prospective studies using standardized ultrasound acquisition, structured neurological assessment, and an appropriate comparator cohort are needed to determine its independent contribution to surgical outcomes.

## Figures and Tables

**Table 1:** Patient and lesion characteristics.

Variable	Value
**Cohort**	
Patients, *n*	15
Age, median (range), years	62 (33–79)
Sex, *n* (%)	
Male	10 (66.7)
Female	5 (33.3)
Primary tumor histology, *n* (%)	
Lung cancer	10 (66.7)
Breast carcinoma	2 (13.3)
Gastrointestinal/colorectal carcinoma	2 (13.3)
Endometrial carcinoma	1 (6.7)
Brain metastasis status, *n* (%)	
Solitary brain metastasis	8 (53.3)
Multiple brain metastases	7 (46.7)
**Operated target lesion**	
Target lesion side, *n* (%)	
Right	9 (60.0)
Left	6 (40.0)
Perirolandic location, *n* (%)	
Precentral gyrus	9 (60.0)
Postcentral gyrus	3 (20.0)
Paracentral lobule	3 (20.0)
Target tumor volume, median (range), cm^3^	8.13 (2.23–55.94)
**Preoperative clinical status**	
Preoperative KPS, median (range)	90 (50–100)
Preoperative motor deficit, *n* (%)	11 (73.3)
Preoperative seizure, *n* (%)	4 (26.7)

Abbreviations: KPS, Karnofsky Performance Status. Percentages are calculated with denominator *n* = 15 unless otherwise indicated.

**Table 2:** Intraoperative ultrasonographic workflow and extent of resection.

Variable	Value
**Image-guided workflow**	
Transdural B-mode iUS before dural opening	15 (100.0)
Repeat B-mode iUS after dural opening	15 (100.0)
Adequate craniotomy coverage confirmed by transdural iUS	15 (100.0)
Secondary craniotomy enlargement	0 (0.0)
Cottonoid-guided iUS	5 (33.3)
Transsulcal corridor among cottonoid-guided cases	3/5 (60.0)
Interhemispheric corridor among cottonoid-guided cases	2/5 (40.0)
Post-resection cavity iUS	14 (93.3)
Epidural iUS after dural closure	5 (33.3)
**Extent of resection**	
Gross-total resection	13 (86.7)
Non-gross-total resection	2 (13.3)

Abbreviations: GTR, gross-total resection; iUS, intraoperative ultrasonography. Percentages are calculated with denominator *n* = 15 unless a different denominator is shown.

**Table 3:** Patient-level comparison of post-resection cavity B-mode intraoperative ultrasonography findings with residual enhancing tumor on early postoperative contrast-enhanced MRI as the reference standard (*n* = 14).

	MRI: Residual Tumor	MRI: No Residual Tumor
iUS: residual suspected	1	6
iUS: no residual	1	6
**Total**	**2**	**12**

Post-resection cavity ultrasonography was performed in 14 of 15 patients; in 1 patient it was not performed because of intraoperative atrial fibrillation. Derived measures, provided for completeness only and not as definitive estimates of diagnostic accuracy: sensitivity 50.0% (95% CI, 9.5–90.5); specificity 50.0% (95% CI, 25.4–74.6); positive predictive value 14.3% (95% CI, 2.6–51.3); negative predictive value 85.7% (95% CI, 48.7–97.4). Confidence intervals were calculated using the Wilson score method. Abbreviations: CI, confidence interval; iUS, intraoperative ultrasonography; MRI, magnetic resonance imaging.

**Table 4:** Early postoperative imaging and clinical outcomes.

Variable	Value
**Early postoperative imaging**	
Mild blood products within the resection cavity on early CT	3 (20.0)
Blood products associated with neurological deterioration	0 (0.0)
Reoperation	0 (0.0)
**Early neurological outcomes**	
New or worsened postoperative motor deficit	2 (13.3)
Partial improvement of motor deficit at 1 month	1 (6.7)
Persistent motor deficit at last follow-up	1 (6.7)
Improved or partially improved motor status	5 (33.3)
Stable motor status without new worsening	8 (53.3)
No postoperative motor worsening	13 (86.7)
Early postoperative seizure	2 (13.3)
Generalized tonic-clonic seizure	1 (6.7)
Seizures controlled with medication at follow-up	2 (13.3)
**Perioperative and hospital outcomes**	
30-day mortality	0 (0.0)
Length of hospital stay, median (range), days	6 (5–14)

All patients had a minimum clinical follow-up of 1 month. Abbreviations: CT, computed tomography. Percentages are calculated with denominator *n* = 15 unless a different denominator is shown.

## Data Availability

The data presented in this study are available on request from the corresponding author due to privacy and ethical restrictions related to patient information.

## Abbreviations

The following abbreviations are used in this manuscript:

2D	two-dimensional
3D	three-dimensional
BM	brain metastasis/metastases
CT	computed tomography
GTR	gross-total resection
iMRI	intraoperative magnetic resonance imaging
IONM	intraoperative neurophysiological monitoring
iUS	intraoperative ultrasonography
KPS	Karnofsky Performance Status
MRC	Medical Research Council
MRI	magnetic resonance imaging
non-GTR	non-gross-total resection

## References

[1] Parker M., Jiang K., Rincon-Torroella J., Materi J., Azad T.D., Kamson D.O., Kleinberg L.R., Bettegowda C. (2023). Epidemiological trends, prognostic factors, and survival outcomes of synchronous brain metastases from 2015 to 2019: A population-based study. Neuro-Oncol. Adv..

[2] Hatiboglu M.A., Wildrick D.M., Sawaya R. (2013). The role of surgical resection in patients with brain metastases. Ecancermedicalscience.

[3] Zuo F., Hu K., Kong J., Zhang Y., Wan J. (2020). Surgical Management of Brain Metastases in the Perirolandic Region. Front. Oncol..

[4] Sanmillan J.L., Fernández-Coello A., Fernández-Conejero I., Plans G., Gabarrós A. (2017). Functional approach using intraoperative brain mapping and neurophysiological monitoring for the surgical treatment of brain metastases in the central region. J. Neurosurg..

[5] Winther R.R., Hjermstad M.J., Skovlund E., Aass N., Helseth E., Kaasa S., Yri O.E., Vik-Mo E.O. (2022). Surgery for brain metastases—impact of the extent of resection. Acta Neurochir..

[6] Hendrix P., Senger S., Griessenauer C.J., Simgen A., Schwerdtfeger K., Oertel J. (2016). Preoperative navigated transcranial magnetic stimulation in patients with motor eloquent lesions with emphasis on metastasis. Clin. Anat..

[7] Krieg S.M., Sabih J., Bulubasova L., Obermueller T., Negwer C., Janssen I., Shiban E., Meyer B., Ringel F. (2014). Preoperative motor mapping by navigated transcranial magnetic brain stimulation improves outcome for motor eloquent lesions. Neuro-Oncology.

[8] Pelletier J.-B., Moiraghi A., Zanello M., Roux A., Peeters S., Trancart B., Edjlali M., Lechapt E., Tauziede-Espariat A., Zah-Bi G. (2021). Is function-based resection using intraoperative awake brain mapping feasible and safe for solitary brain metastases within eloquent areas?. Neurosurg. Rev..

[9] Kaale A.J., Rutabasibwa N., Mchome L.L., Lillehei K.O., Honce J.M., Kahamba J., Ormond D.R. (2021). The use of intraoperative neurosurgical ultrasound for surgical navigation in low- and middle-income countries: The initial experience in Tanzania. J. Neurosurg..

[10] Dixon L., Lim A., Grech-Sollars M., Nandi D., Camp S. (2022). Intraoperative ultrasound in brain tumor surgery: A review and implementation guide. Neurosurg. Rev..

[11] Sirbu O.M., Chirtes A., Gorgan M.R., Mitrica M. (2025). 2D Intraoperative Ultrasound in Brain Metastasis Resection: A Matched Cohort Analysis from a Single-Center Experience. Cancers.

[12] Elmesallamy W.A.E.A. (2019). The role of intraoperative ultrasound in gross total resection of brain mass lesions and outcome. Egypt. J. Neurol. Psychiatry Neurosurg..

[13] Di Cristofori A., Carone G., Rocca A., Rui C.B., Trezza A., Carrabba G., Giussani C. (2023). Fluorescence and Intraoperative Ultrasound as Surgical Adjuncts for Brain Metastases Resection: What Do We Know? A Systematic Review of the Literature. Cancers.

[14] Frigeri T., Paglioli E., de Oliveira E., Rhoton A.L. (2015). Microsurgical anatomy of the central lobe. J. Neurosurg..

[15] Keles A., Ture U. (2022). Cottonoid-guided intraoperative ultrasonography in neurosurgery: A proof-of-concept single surgeon case series. Neurosurg. Rev..

[16] Dogruel Y., Rahmanov S., Gungor A., Ture U. (2023). Opening the Parieto-Occipital Fissure for Periatrial Metastasis: 2-Dimensional Operative Video. Oper. Neurosurg..

[17] Saß B., Carl B., Pojskic M., Nimsky C., Bopp M. (2020). Navigated 3D Ultrasound in Brain Metastasis Surgery: Analyzing the Differences in Object Appearances in Ultrasound and Magnetic Resonance Imaging. Appl. Sci..

[18] Magill S.T., Han S.J., Li J., Berger M.S. (2018). Resection of primary motor cortex tumors: Feasibility and surgical outcomes. J. Neurosurg..

[19] Bander E.D., Shelkov E., Modik O., Kandula P., Karceski S.C., Ramakrishna R. (2020). Use of the train-of-five bipolar technique to provide reliable, spatially accurate motor cortex identification in asleep patients. Neurosurg. Focus.

[20] Willems P.W.A., van der Sprenkel J.W.B., Tulleken C.A.F., Viergever M.A., Taphoorn M.J.B. (2006). Neuronavigation and surgery of intracerebral tumours. J. Neurol..

[21] Munkvold B.K.R., Jakola A.S., Reinertsen I., Sagberg L.M., Unsgård G., Solheim O. (2018). The Diagnostic Properties of Intraoperative Ultrasound in Glioma Surgery and Factors Associated with Gross Total Tumor Resection. World Neurosurg..

[22] Brown P.D., Ballman K.V., Cerhan J.H., Anderson S.K., Carrero X.W., Whitton A.C., Greenspoon J., Parney I.F., Laack N.N.I., Ashman J.B. (2017). Postoperative stereotactic radiosurgery compared with whole brain radiotherapy for resected metastatic brain disease (NCCTG N107C/CEC·3): A multicentre, randomised, controlled, phase 3 trial. Lancet Oncol..

[23] Mahajan A., Ahmed S., McAleer M.F., Weinberg J.S., Li J., Brown P., Settle S., Prabhu S.S., Lang F.F., Levine N. (2017). Post-operative stereotactic radiosurgery versus observation for completely resected brain metastases: A single-centre, randomised, controlled, phase 3 trial. Lancet Oncol..

[24] Patchell R.A., Tibbs P.A., Regine W.F., Dempsey R.J., Mohiuddin M., Kryscio R.J., Markesbery W.R., Foon K.A., Young B. (1998). Postoperative Radiotherapy in the Treatment of Single Metastases to the Brain: A Randomized Trial. JAMA.

[25] Chan H.W., Uff C., Chakraborty A., Dorward N., Bamber J.C. (2021). Clinical Application of Shear Wave Elastography for Assisting Brain Tumor Resection. Front. Oncol..

[26] Prada F., Bene M.D., Fornaro R., Vetrano I.G., Martegani A., Aiani L., Sconfienza L.M., Mauri G., Solbiati L., Pollo B. (2016). Identification of residual tumor with intraoperative contrast-enhanced ultrasound during glioblastoma resection. Neurosurg. Focus.

[27] Cheng L., Wang F., Yin L., Zhang L., Kang R., Zhang W., He W. (2026). Application of Intraoperative Conventional and Contrast-Enhanced Ultrasound in Brain Metastases: A Preliminary Study. J. Ultrasound Med..

[28] Serra C., Stauffer A., Actor B., Burkhardt J.K., Ulrich N.H., Bernays R.L., Bozinov O. (2012). Intraoperative high frequency ultrasound in intracerebral high-grade tumors. Ultraschall Med..

